# Historical survey of the *kdr* mutations in the populations of *Anopheles sinensis* in China in 1996–2014

**DOI:** 10.1186/s12936-015-0644-0

**Published:** 2015-03-20

**Authors:** Yan Wang, Wanqin Yu, Hua Shi, Zhenzhou Yang, Jiannong Xu, Yajun Ma

**Affiliations:** Department of Tropical Infectious Diseases, Second Military Medical University, Shanghai, 200433 China; Center for Disease Control and Prevention of P. L.A., Beijing, 100071 China; Department of Biology, Molecular Biology Program, New Mexico State University, Las Cruces, NM 88003 USA

**Keywords:** *Anopheles sinensis*, *Anopheles lesteri*, Pyrethroids, Knockdown resistance, *Kdr* allele

## Abstract

**Background:**

*Anopheles sinensis* has become an important malaria vector in China. The long-term extensive utilization of pyrethroids for ITNs and IRS for mosquito control in the last three decades has resulted in the occurrence of resistant *An. sinensis* populations in many regions. Knockdown resistance (*kdr*), caused by point mutations in the VGSC gene, is one of the mechanisms that confer resistance to DDT and pyrethroids. Recently, several investigations revealed the *kdr* occurrence in some *An. sinensis* populations, however, no *kdr* data were available earlier than 2009. A survey tracking the dynamics of the *kdr* mutations in past decades would provide invaluable information to understand how the *kdr* alleles spread in mosquito populations temporally and spatially.

**Methods:**

A survey was conducted on the *kdr* alleles at condon 1014 of the VGSC gene and their distributions in 733 specimens of *An. sinensis* and 232 specimens of the other eight member species of the *Anopheles hyrcanus* group that were collected from 17 provinces in China in 1996–2014.

**Results:**

A total of three *kdr* alleles, TTT (F), TTG (F) and TGT (C) were detected, and TGT (C) and TTT (F) were already present in the specimens from Jiangsu and Shandong as early as 1997. The TTT (F) was the most frequent mutant allele, and largely distributed in central China, namely Shandong, Jiangsu, Anhui, Henan, Shanghai, Jiangxi and Hubei. When data were analysed in three time intervals, 1996–2001, 2005–2009, 2010–2014, the prevalence of *kdr* alleles increased progressively over time in the populations in central China. In contrast, the *kdr* alleles were less frequent in the samples from other regions, especially in Yunnan and Hainan, despite the documented presence of pyrethroid resistant populations in those regions. Interestingly, no mutant alleles were detected in all 232 specimens of eight other species in the *An. hyrcanus* group.

**Conclusion:**

The survey revealed that the *kdr* occurrence and accumulation in the *An. sinensis* populations were more frequent in central China than in the other regions, suggesting that the *kdr* mutations may contribute significantly to the pyrethroid resistance in the mosquitoes in central China.

**Electronic supplementary material:**

The online version of this article (doi:10.1186/s12936-015-0644-0) contains supplementary material, which is available to authorized users.

## Background

Mosquito control is one of the integrated programmes to prevent transmission of mosquito-borne diseases such as malaria, filariasis, and dengue fever. Chemical insecticides have been extensively used for vector management since the 1940s. Four major categories of insecticides have been utilized: organochlorines, organophosphates, carbamates and pyrethroids [1]. DDT and pyrethroids function as neurotoxins that target voltage-gated sodium channels (VGSC) and interfere electronic signaling in the nervous system, which results in paralysis and death, an effect known as knockdown [2]. One of the mechanisms that mosquitoes have developed for the resistance to DDT and pyrethroids is the target insensitivity, which is caused by mutations in the VGSC gene. A prominent mutation is the substitution of leucine at residue position 1014 in mosquitoes with the knockdown resistance (*kdr*) [1]. A positive correlation between the *kdr* mutation and the resistant phenotype to pyrethroids and DDT was well documented in various *Anopheles* populations [3-10].

*Anopheles sinensis* is an Oriental species with wide distributions in China. *Anopheles sinensis* is one of the principal malaria vectors in many malaria-endemic regions, especially in the central China, due to its abundant population size [11]. In China, DDT has been widely used for conventional indoor residue sprays (IRS) since 1950s, and pyrethroids have been applied for IRS and insecticide-treated nets (ITNs) since 1980s [12]. These measures have been effective in reducing malaria transmission [13]. However, the long-term applications of insecticides have resulted in the development of resistance in mosquito populations. For instance, the DDT resistance in *An. sinensis* was documented in Yunnan as early as 1981 [14]; the permethrin resistance was reported in Sichuan in 1989 [15]; and in Fujian in 1989–1993, resistant populations occurred two years after IRS and ITNs applications, and the resistance spread in more populations three years after applications [13].

Recent years, the *kdr* genotyping has been included in monitoring pyrethroid resistance in *An. sinensis* in China*.* Several investigations have been made on the distributions of *kdr* alleles in various *An. sinensis* populations, such as provinces Jiangsu, Henan, Hunan, Anhui, Jiangxi, Yunnan, and Hainan [7,16-21]. However, these studies were conducted in the last five years, the *kdr* data were obtained from the mosquito specimens that were sampled in 2009 and after. No earlier *kdr* data were available. A historical survey tracking the dynamics of the *kdr* mutations in the past decades would provide invaluable information to understand how the *kdr* mutations occurred and spread in mosquito populations temporally and spatially. Therefore, a study was conducted to investigate the genotypes of the codon 1014 of the VGSC gene in the specimens of *An. sinensis* and the other eight member species of the *An. hyrcanus* group that were collected from 17 provinces in 1996–2014. The data revealed that the *kdr* alleles were already present in the specimens sampled in 1997, and *kdr* alleles progressively increased over decades in the *An. sinensis* populations in central China. The *kdr* were much less prevalent in the other populations, particularly in Yunnan and Hainan.

## Methods

### Mosquito collections and species identification

**Figure 1:**
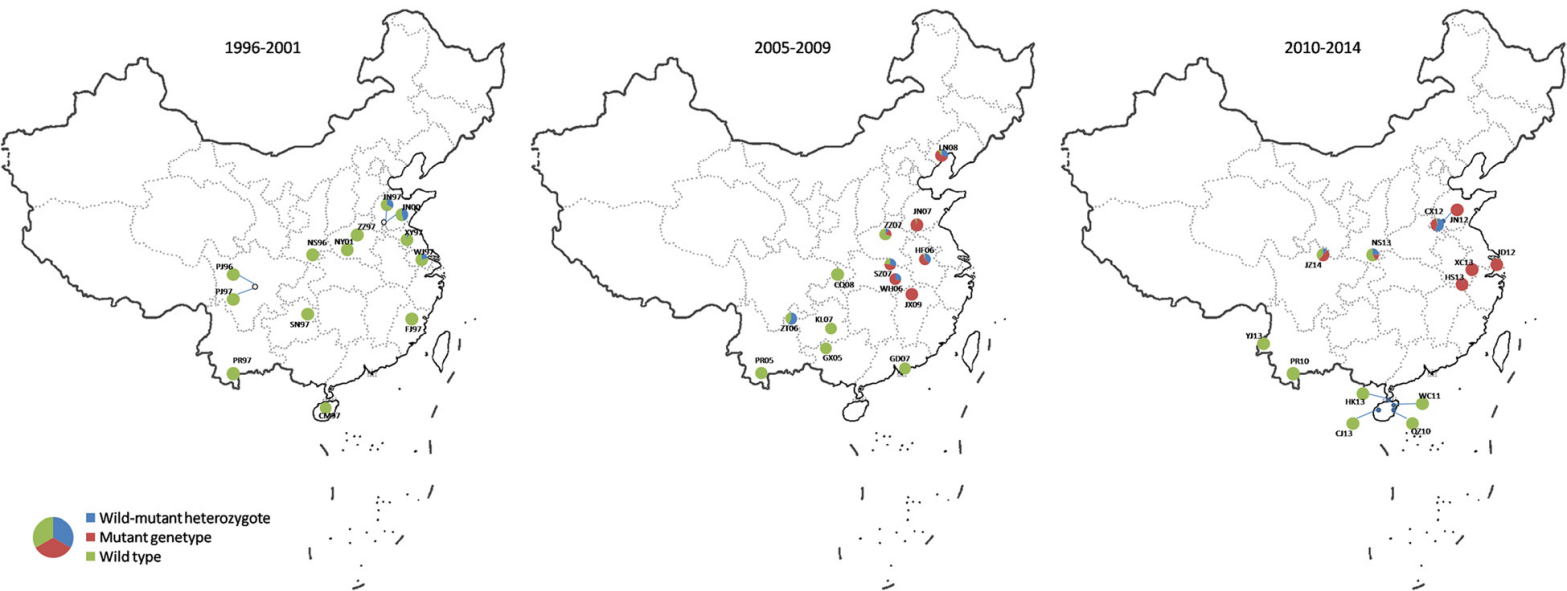
**A schematic map of sampling sites for** ***Anopheles sinensis*** **in China and the composition of the** ***kdr*** **genotypes in the samples in the three time intervals, 1996–2001, 2005–2009 and 2010–2014.** The genotype composition in each location was displayed by a pie chart with blue as wild/mutant heterozygote, red as mutant genotype and green as wildtype genotype (see Additional file 2: Table S2 for details).

Wild mosquito adults were collected in 1996–2014 from 31 sampling sites in 17 provinces in China. Mosquitoes were caught by using light traps at livestock corrals or human landing catches, with consent of the owners and persons involved in the study. The collection information was summarized in Tables 1, 2 and Figure 1. Mosquitoes of the *Anopheles hyrcanus* group were sorted out in the field by morphology using the identification keys [11], and brought back to the lab. The species identity was molecularly determined by a diagnostic PCR assay based on the ribosomal DNA (rDNA) second internal transcribed spacer (ITS2) markers [22] or by the ITS2 sequencing [23].

**Table 1:** **Sampling information of** ***Anopheles sinensis*** **in China**

**Collection site**		**Latitude/longitude coordinates**	**Code**	**Sample size**	**Date**
Anhui	Hefei	N:31°52′, E:117°16′	HF06	19	07/2006
	Huangshan	N:30°08′, E:118°10′	HS13	60	07/2013
	Xuancheng	N:30°57′, E:118°44′	XC13	30	07/2013
Chongqing	Kaixian	N:31°10′, E:108°23′	CQ08	16	07/2008
Fujian	Jianyang	N:27°24′, E:118°03′	FJ97	20	09/1997
Guangdong	Zhuhai	N:22°15′, E:113°33′	GD07	26	10/2007
Guangxi	Tiane	N:25°01′, E:106°59′	GX05	22	07/2005
Guizhou	Sinan	N:27°51′, E:108°08′	SN97	16	05/1997
	Kaili	N:26°37′, E:107°56′	KL07	20	08/2007
Hainan	Chengmai	N:19°45′, E:110°00′	CM97	7	05/1997
	Qiongzhong	N:19°03′, E:109°50′	QZ10	12	08/2010
	Wenchang	N:19°36′, E:110°43′	WC11	14	08/2011
	Changjiang	N:19°15′, E:109°02′	CJ13	7	06/2013
	Haikou	N:20°01′, E:110°20′	HK13	5	08/2013
Henan	Zhengzhou	N:34°45′, E:113°38′	ZZ97	9	07/1997
			ZZ07	10	08/2007
	Nanyang	N:33°00′, E:112°31′	NY01	23	06/2001
Hubei	Wuhan	N:30°34′, E:114°18′	WH06	24	08/2006
	Suizhou	N:31°43′, E:113°22′	SZ07	13	07/2007
Jiangsu	Xuyi	N:32°58′, E:118°31′	XY97	20	07/1997
	Wujing	N:31°40, E:119°56′	WJ97	24	07/1997
Jiangxi	Yongxiu	N:29°08′, E:115°44′	JX09	16	09/2009
Liaoning	Huludao	N:40°44′, E:120°51′	LN08	10	08/2008
Shandong	Jining	N:35°24′, E:116°35	JN97	3	07/1997
			JN00	31	07/2000
			JN07	22	08/2007
			JN12	12	07/2012
	Caoxian	N:34°49′, E:115°32′	CX12	30	07/2012
Shanghai	Jiading	N:31°22′, E:121°14′	JD12	24	08/2012
Shaanxi	Ningshan	N:33°32′, E:108°26′	NS96	2	08/1996
			NS13	36	07/2013
Sichuan	Pujiang	N:30°14′, E:102°29′	PJ96	20	07/1997
			PJ97	18	07/1997
	Jiuzhaigou	N:33°16′, E:104°14′	JZ14	46	07/2014
Yunnan	Puer	N:22°47′, E:100°58′	PR97	10	05/1997
			PR05	10	08/2005
			PR10	17	07/2010
	Zhaotong	N:27°20′, E:103°43′	ZT06	14	07/2006
	Yingjiang	N:24°51′, E:97°55′	YJ13	15	08/2013

**Table 2:** **Sampling information of member species in the** ***Anopheles hyrcanus*** **group in China**

**Collection site**		**Latitude/longitude coordinates**	**Species**	**Sample size**	**Date**
Guangdong	Zhuhai	N:22°15′, E:113°33′	*An. lesteri*	2	07/2001
				9	10/2007
Guangxi	Lab colony	*--*	*An. lesteri*	1	-
Hainan	Wenchang	N:19°36′, E:110°43′	*An. lesteri*	11	12/2001
				6	08/2010
Henan	Nanyang	N:33°00′, E:112°31′	*An. lesteri*	5	06/2001
			*An. yatsushiroensis*	3	
Hubei	Suizhou	N:31°43′, E:113°22′	*An. lesteri*	4	07/2007
Jiangsu	Lab colony	*--*	*An. lesteri*	17	-
Liaoning	Zhangwu	N:42°31′, E:122°28′	*An. lesteri*	1	08/2007
	Zhuanghe	N:39°51′, E:122°55′	*An. lesteri*	2	08/1999
	Faku	N:42°24′, E:123°14′	*An. lesteri*	1	08/1996
				12	08/1998
			*An. yatsushiroensis*	5	
	Sujiatun	N:41°34′, E:123°25′	*An. lesteri*	1	08/1997
				8	08/1998
				2	08/2002
			*An. yatsushiroensis*	1	
	Donggang	N:39°58′, E:123°52′	*An. lesteri*	18	07/1997
			*An. yatsushiroensis*	6	
			*An. lesteri*	11	07/1999
			*An. yatsushiroensis*	1	
			*An. lesteri*	44	08/2004
			*An. yatsushiroensis*	3	
Shandong	Rongchen	N:37°10′, E:122°29′	*An. yatsushiroensis*	3	08/2004
	Jining	N:35°24′, E:116°35	*An. belenrae*	3	08/1997
				7	08/2012
	Caoxian	N:34°49′, E:115°32′	*An. belenrae*	3	08/2012
Shaanxi	Yulin	N:38°16′, E:109°44′	*An. keleini*	16	08/2009
				3	08/2013
Sichuan	Junlian	N:28°02′, E:104°35′	*An. lesteri*	6	07/1996
	Pujiang	N:30°14′, E:102°29′	*An. lesteri*	2	07/1998
			*An. yatsushiroensis*	2	
	Liangshan	N:27°53′, E:102°35′	*An. liangshanensis*	3	08/1997
Yunnan	Zhaotong	N:27°20′, E:103°43′	*An. lesteri*	2	08/1999
				1	08/2006
	Kunming	N:25°03′, E:102°42′	*An. kunmingensis*	2	08/1997
	Puer	N:22°47′, E:100°58′	*An. lesteri*	1	08/1999
			*An. peditaeniatus*	2	08/2007
			*An. crawfordi*	2

### *kdr* gene amplification and sequencing

To identify *kdr* alleles, a partial sequence of S6 segment of domain II of the VGSC gene was amplified from 20-50 ng genomic DNA from single specimens using CD1 (5′-TGA TCG TGT TTC GCG TGC TG-3′) and CD2 (5′-GTC TCG TTA TCC GCC GTT GG-3′) primers [3]. The PCR kit was from Aidlab, China. The PCR reaction was carried out in Verity 96 well Thermal Cycler (Applied Biosystems, USA) included an initial step of denaturation at 94°C for 1 min, followed by 35 cycles of amplification at 94°C for 30 s, 55°C for 30 s, and 68°C for 30 s, with a final extension step at 68°C for 7 min. After electrophoresis, PCR products were purified and used for sequencing in both directions with the CD1 and CD2 primers, respectively. There were 37 specimens, of which the PCR products were cloned into plasmids (pGEMX-T Easy Vector, Aidlab, China), and then sequenced, due to the double peaks at two positions of the codon 1014.

### Statistical analyses

The codon 1014 was examined by sequence analysis, and genotypes were determined. In each sample, for a particular allele, the allele frequency was calculated as: number of alleles/(sample size × 2). The mutation frequency was defined as frequency of sum of wildtype/mutant heterozygotes and mutant/mutant homozygotes, which was calculated as: (sum of wildtype/mutant and mutant/mutant individuals)/sample size. The maximum likely frequency (y) of an allele present or absent in a sample of a given size (x) was obtained from the upper of 95% confidence limit of binomial distribution, given by y = 1–0.051/x, following the example of Post and Millest [24].

## Results

### Taxonomic composition of the mosquito collections

In the current study, 733 specimens of *An. sinensis* were used in the *kdr* allele survey. Some of these samples were from mosquito collections of our previous work on the molecular identification and phylogeny of the *An. hyrcanus* group [22,23] and the population genetics of *An. sinensis* [25] and *Anopheles lesteri* [26] since 1996. The samples included specimens from 31 sampling sites in 17 provinces in China from 1996 to 2014 (Figure 1 and Table 1). In addition, 232 mosquitoes of eight other species in the *An. hyrcanus* group were identified in the samples collected in 19 locations from 11 provinces in China in 1996–2013, including 167 of *Anopheles lesteri*, 24 of *Anopheles yatsushiroensis,* 13 of *Anopheles belenrae*, 19 of *Anopheles kleini*, three of *Anopheles liangshanensis*, two of *Anopheles peditaeniatus*, two of *Anopheles kunmingensis* and two of *Anopheles crawfordi*.

### Frequency and distribution of *kdr* mutations in the *An. sinensis* populations

In order to detect *kdr* alleles, a 343 bp fragment of the IIS6 domain of the VGSC gene was PCR amplified and sequenced directly. At codon 1014, four alleles were identified in the *An. sinensis* samples. In addition to the wildtype codon TTG encoding leucine (L), three mutant alleles were detected. Codons TTT and TTC both code for phenylalanine (F), and codon TGT codes for cysteine (C). A total of 10 genotypes were detected, including wildtype homozygote TTG/TTG (55.25%), mutant homozygotes TTT/TTT (17.60%), TTC/TTC (0.27%), TGT/TGT (1.91%), wildtype/mutant heterozygotes TTG/TTT (8.19%), TTG/TTC (0.82%), TTG/TGT (4.50%), TTT/TTC (0.68%), TTT/TGT (10.23%), and TTC/TGT (0.55%). Overall, the frequency of mutant genotypes (F/F, C/C and F/C) accounted for 31.24%, and the frequency of heterozygote genotypes L/F and L/C was 13.51%. The *kdr* allele frequency was presented in Additional file 1: Table S1.The geographic distribution of genotypes, i.e. wildtype homozygotes, wildtype/mutant heterozygotes, and mutant homozygotes, were depicted in Figure 1. The mutant alleles (1014F and 1014C) were highly prevalent in seven regions in central China, namely Shandong, Henan, Jiangsu, Shanghai, Anhui, Jiangxi and Hubei. The mutant alleles were less prevalent in the samples from other regions, including Sichuan, Chongqing, Shaanxi, Yunnan, Guizhou, and Hainan Island (Additional file 1: Table S1).

The specimens were collected in a time span of 1996–2014. To trace back to the temporal occurrence of *kdr* genotypes, the data were analysed in three time intervals, 1996–2001, 2005–2009 and 2010–2014 (Additional file 2: Table S2). In1996-2001, 203 specimens in 13 samples from 10 provinces were examined, the mutant alleles TGT (C) and TTT (F) were detected in 21 specimens, and seven of these specimens were found in Shandong and Jiangsu as early as 1997. These specimens were all wildtype/mutant heterozygotes, L/F and L/C. The mutation frequency was 5.0-45.16% in the populations of 1996–2001 (Additional file 2: Table S2). In 2005–2009, 222 specimens in 13 samples from 11 provinces were screened, three mutant genotypes, TTT/TTT, TGT/TGT, and TTC/TGT, occurred with frequency of 0.45-21.62% in the seven samples from six provinces, Shandong, Henan, Anhui, Hubei, Jiangxi, and Liaoning. The mutation frequency rose to 30-100%. No mutant alleles were detected in the samples from the other five regions (Additional file 1: Table S1 and Additional file 2: Table S2). In 2010–2014, 308 specimens in 13 samples from seven regions were investigated. The *kdr* alleles were found in Shandong, Shanghai, Anhui, Shaanxi, and Sichuan (Additional file 1: Table S1 and Additional file: 2: Table S2). In addition to the mutant genotypes mentioned above, two more mutant genotypes, TTC/TTC and TTT/TTC, were detected in Anhui and Sichuan. The mutation frequency was 41.67-100%. No mutant alleles were detected in the samples from Yunnan and Hainan (Additional file 2: Table S2).

**Figure 2:**
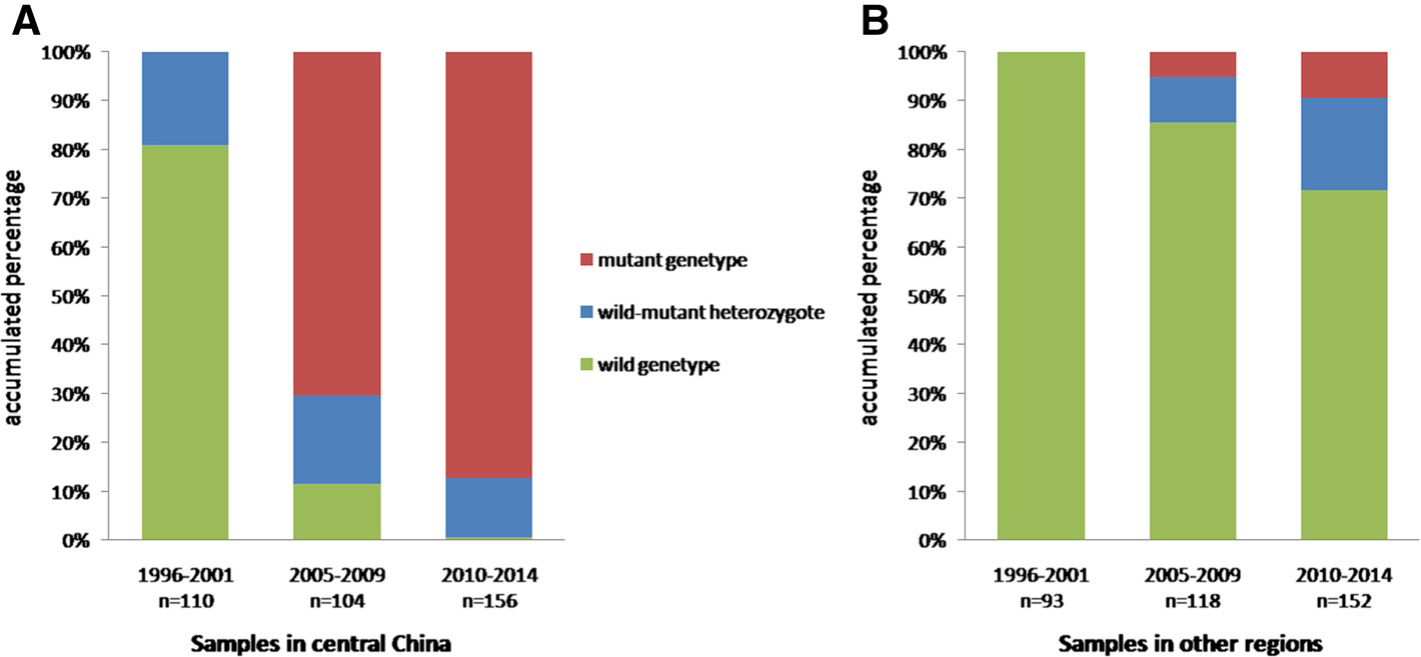
**Temporal and spatial trend of** ***kdr*** **genotypes in** ***Anopheles sinensis*** **in China in 1997–2014. (A)** Mosquitoes were sampled in Shandong, Jiangsu, Anhui, Shanghai, Hubei, Henan and Jiangxi (data in Table 3). **(B)** Mosquitoes were sampled in Liaoning, Shaanxi, Guizhou, Sichuan, Chongqing, Yunnan, Hainan, Fujian, Guangdong, and Guangxi (data in Table 4). See Figure 1 for sampling sites on the map.

Geographically, the *kdr* alleles were distributed largely in the populations from central China. Therefore, the samples from central China were further analysed. As shown in Table 3, the frequency of wildtype genotype dropped over time, from 80.91% in 1996–2001 to 0.64% in 2010–2014, whilst the frequency of mutant genotypes rose from 0 to 87.18% (Table 3, Figure 2A). Furthermore, the mutant genotypes F/F and F/C were enriched in Shandong, Shanghai and Anhui in 2012–2014 (Table 3). In the regions other than central China, no mutant alleles were detected in the three of four samples from Yunnan (1996–2013) and five samples from Hainan (1997, 2010–2013), and two samples from Guizhou (1997, 2007), one sample from Guangdong (2007), one sample from Fujian (1997) and one sample from Guangxi (2005) (Table 4). Overall, the frequency of wildtype homozygotes was 100% in 1997–2001, and still relatively high as 71.71% in 2010–2014 in these regions (Figure 1, Figure 2B). The analysis revealed that the *kdr* alleles were enriched in the *An. sinensis* populations in central China with a trend of progressive increase over time. The *kdr* alleles were less prevalent in the other regions of China.

**Table 3:** **The frequency of** ***kdr*** **mutations in the** ***Anopheles sinensis*** **samples in central China**

**Sample code**	**Sample location**	**Sample size**	**Wildtype**	**Wildtype/mutant heterozygote**			**Mutant genotype**						**Mutation frequency (%)**
							**Mutant homozygote**			**Mutant heterozygote**			
			**TTG(L)/ TTG(L)**	**TTG(L)/ TTT(F)**	**TTG(L)/ TTC(F)**	**TTG(L)/ TGT(C)**	**TTT(F)/ TTT(F)**	**TTC(F)/ TTC(F)**	**TGT(C)/ TGT(C)**	**TTT(F)/ TTC(F)**	**TTT(F)/ TGT(C)**	**TTC(F)/ TGT(C)**	
1997-2001													
ZZ97	Henan	9	9	0	0	0	0	0	0	0	0	0	0.00
XY97	Jiangsu	20	19	0	0	1	0	0	0	0	0	0	5.00
WJ97	Jiangsu	24	19	3	0	2	0	0	0	0	0	0	20.83
JN97	Shandong	3	2	0	0	1	0	0	0	0	0	0	33.33
JN00	Shandong	31	17	4	0	10	0	0	0	0	0	0	45.16
NY01	Henan	23	23	0	0	0	0	0	0	0	0	0	0.00
Total		110	89	7	0	14	0	0	0	0	0	0	19.09
Genotype frequency(%)			80.91	6.36	0.00	12.73	0.00	0.00	0.00	0.00	0.00	0.00	
2005-2009													
HF06	Anhui	19	1	4	0	3	8	0	0	0	3	0	94.74
WH06	Hubei	24	0	3	0	4	5	0	5	0	7	0	100.00
SZ07	Hubei	13	3	4	0	0	2	0	0	0	4	0	76.92
ZZ07	Henan	10	7	0	0	1	0	0	0	0	2	0	30.00
JN07	Shandong	22	1	0	0	0	16	0	0	0	5	0	95.45
JX09	Jiangxi	16	0	0	0	0	12	0	0	0	3	1	100.00
Total		104	12	11	0	8	43	0	5	0	24	1	88.46
Genotype frequency(%)			11.54	10.58	0.00	7.69	41.35	0.00	4.81	0.00	23.08	0.96	
2010-2014													
JN12	Shandong	12	0	0	0	0	7	0	1	0	4	0	100.00
CX12	Shandong	30	1	11	0	6	10	0	0	0	2	0	96.67
JD12	Shanghai	24	0	0	0	0	12	0	1	0	10	1	100.00
HS13	Anhui	60	0	2	0	0	28	1	4	3	20	2	100.00
XC13	Anhui	30	0	0	0	0	15	0	2	0	13	0	100.00
Total		156	1	13	0	6	72	1	8	3	49	3	99.36
Genotype frequency(%)			0.64	8.33	0.00	3.85	46.15	0.64	5.13	1.92	31.41	1.92

**Table 4:** **The frequency of** ***kdr*** **mutations in the** ***Anopheles sinensis*** **samples in other regions**

**Sample code**	**Sample location**	**Sample size**	**Wildtype**	**Wildtype/mutant heterozygote**			**Mutant genotype**						**Mutation frequency (%)**
							**Mutant homozygote**			**Mutant heterozygote**			
			**TTG(L)/ TTG(L)**	**TTG(L)/ TTT(F)**	**TTG(L)/ TTC(F)**	**TTG(L)/ TGT(C)**	**TTT(F)/ TTT(F)**	**TTC(F)/ TTC(F)**	**TGT(C)/ TGT(C)**	**TTT(F)/ TTC(F)**	**TTT(F)/ TGT(C)**	**TTC(F)/ TGT(C)**	
1997-2001													
NS96	Shaanxi	2	2	0	0	0	0	0	0	0	0	0	0.00
PJ96	Sichuan	20	20	0	0	0	0	0	0	0	0	0	0.00
SN97	Guizhou	16	16	0	0	0	0	0	0	0	0	0	0.00
PR97	Yunnan	10	10	0	0	0	0	0	0	0	0	0	0.00
CM97	Hainan	7	7	0	0	0	0	0	0	0	0	0	0.00
PJ97	Sichuan	18	18	0	0	0	0	0	0	0	0	0	0.00
FJ97	Fujian	20	20	0	0	0	0	0	0	0	0	0	0.00
Total		93	93	0	0	0	0	0	0	0	0	0	0.00
Genotype frequency(%)			100.00	0.00	0.00	0.00	0.00	0.00	0.00	0.00	0.00	0.00	
2005-2009													
GX05	Guangxi	22	22	0	0	0	0	0	0	0	0	0	0.00
PR05	Yunnan	10	10	0	0	0	0	0	0	0	0	0	0.00
ZT06	Yunnan	14	6	4	2	2	0	0	0	0	0	0	57.14
KL07	Guizhou	20	20	0	0	0	0	0	0	0	0	0	0.00
GD07	Guangdong	26	26	0	0	0	0	0	0	0	0	0	0.00
CQ08	Chongqing	16	16	0	0	0	0	0	0	0	0	0	0.00
LN08	Liaoning	10	1	2	0	1	5	0	0	0	1	0	90.00
Total		118	101	6	2	3	5	0	0	0	1	0	14.41
Genotype frequency(%)			85.59	5.08	1.69	2.54	4.24	0.00	0.00	0.00	0.85	0.00	
2010-2014													
PR10	Yunnan	17	17	0	0	0	0	0	0	0	0	0	0.00
QZ10	Hainan	12	12	0	0	0	0	0	0	0	0	0	0.00
WC11	Hainan	14	14	0	0	0	0	0	0	0	0	0	0.00
CJ13	Hainan	7	7	0	0	0	0	0	0	0	0	0	0.00
NS13	Shaanxi	36	21	7	1	1	5	0	1	0	0	0	41.67
YJ13	Yunnan	15	15	0	0	0	0	0	0	0	0	0	0.00
HK13	Hainan	5	5	0	0	0	0	0	0	0	0	0	0.00
JZ14	Sichuan	46	18	16	3	1	4	1	0	2	1	0	60.87
Total		152	109	23	4	2	9	1	1	2	1	0	28.29
Genotype frequency(%)			71.71	15.13	2.63	1.32	5.92	0.66	0.66	1.32	0.66	0.00	

### The allele of codon 1014 in other species of the *An. hyrcanus* group

A total of 232 specimens from eight other species in the *An. hyrcanus* group were screened. No *kdr* alleles were detected. All of these specimens were the homozygotes of the wildtype allele TTG.

## Discussion

In China, DDT was largely used in 1950s-1970s, and pyrethroids have been utilized since 1980s when DDT was banned [27]. The applications of pyrethroids for IRS and ITNs have greatly contributed to the success of reducing malarial transmission. The occurrence of pyrethroid resistance in *An. sinensis* has been documented in many regions since 1980s [28,29]. For example, in Zhejiang [30], Hubei [31], Jiangsu [32], Shandong [33], Yunnan [34], Henan [34], Fujian [35], and Hainan [36]. Recently, Wang *et al.* conducted a survey of pyrethroid susceptibility in the *An. sinensis* populations from eight malaria endemic regions including Hubei, Henan, Hunan, Jiangsu, Jianxi, Sichuan, Shanghai and Yunnan [37]. They found that the *An. sinensis* populations in all examined regions were resistant to deltamethrin. The mortalities were in a range of 5.96-64.54% upon exposure to the diagnostic concentration of 0.25% deltamethrin [37]. The relationship of *kdr* genotypes and pyrethroid resistance has been investigated in recent years. At codon 1014 of the VGSC gene, five mutant alleles, TTT(F), TTG(F), TGT(C), TCG(S) and TGG(W), have been detected. The TTT(F) allele was the most prevalent mutant, followed by the alleles TGT(C) and TCG(S); the allele TGG(W) occurred rarely [7,16-19,21,34,38,39]. The *kdr* alleles were present with high frequency largely in the populations in central China, such as Jiangsu, Anhui, Shandong, Hubei, and Henan. For example, Qi and Cui detected the *kdr* alleles 1014F and 1014C in three populations from Henan, and the *kdr* allele frequency was associated with the resistant phenotype. However, no *kdr* alleles were detected in the two populations from Yunnan [34]. In the five populations collected from Jiangsu in 2009–2010, the *kdr* alleles 1014F and 1014C were detected and the frequency of allele 1014F was correlated with the resistance to beta-cypermethrin in these populations [16]. In the Guangxi collections, the *kdr* alleles 1014S, 1014F and 1014W were found [20]. In an investigation reported by Zhong *et al.* the *kdr* alleles 1014F and 1014C were present with high frequency (88.5-94.8%) in the resistant populations from Hunan, Hubei and Jiangsu in central China [17]. Both *kdr* alleles and the monooxygenase activity were significantly associated with the deltamethrin resistance, but the monooxygenase activity played a stronger role. On the other hand, no *kdr* alleles were detected in the two resistant populations in Yunnan, where the resistance was correlated with the monooxygenase activity [17]. A similar pattern was found in another study, in which the *kdr* mutation L1014F (70.0-88.9%) and L1014C (11.1-26.7%) were detected in the Anhui populations with higher frequency in the resistant mosquitoes [19]. Again, no *kdr* alleles were found in the mosquitoes from Yunnan. Based on a CART statistical analysis, metabolic detoxification enzymes (monooxygenases, glutathion S-transferase and carboxylesterases) played major roles in resistance to pyrethroids and DDT while *kdr* alleles weighed less in the context [19]. In Hainan Island, the L/F heterozygotes were present with low frequency (6.7-9.5%) in the DDT and pyrethroid-resistant individuals of the two *An. sinensis* populations, but no *kdr* alleles were detected in the sympatric *An. vagus* [18]. Overall, aforementioned studies demonstrated that the *kdr* mutations occurred largely in the *An. sinensis* populations in central China, where the resistance to DDT and pyrethroids was conferred primarily by the metabolic detoxification mechanisms as well as the *kdr* mutation. In the populations in other regions, the *kdr* alleles were less prevalent, and the pyrethroid resistance was conferred by the metabolic mechanisms.

In the current study, codon 1014 was examined in a large collection of the *An. sinensis* samples covering 17 provinces in a time span of 1996 to 2014. The *kdr* alleles 1014C and 1014F were found in the specimens sampled from Jiangsu and Shandong as early as 1997 (Additional file 1: Table S1). This clearly indicated that the *kdr* alleles already existed in the *An. sinensis* populations in 1990s. In line with the findings in the other reports mentioned above, the *kdr* alleles were more prevalent in central China. The occurrence of *kdr* alleles has been progressively increasing over time (Figures 1 and 2). For example, in the samples from Shandong that were collected in 1997, 2007, and 2012, the mutation frequency increased from 33.3% in 1997, 95.45% in 2007 to 100% in 2012 (Table 3). In the other regions, the *kdr* genotypes frequency was lower in Sichuan (0 in 1997 and 60.78% in 2014). Moreover, no *kdr* alleles were detected in the samples from Yunnan (2005–2013, except ZT06), Guangxi (2005), Fujian (1997), and Hainan (1997–2013) (Table 4). Apparently the pyrethroid resistance in these regions was conferred majorly by the mechanisms other than *kdr* mutations. Similar situations have been reported in *An. gambiae* in Africa. In south-western Nigeria, the *kdr* alleles were not found in the pyrethroid-resistant individuals of the molecular M form of *An. gambiae* [40]. In a survey conducted in south-western Chad, central Africa, the allele 1014F was frequently present in the resistant S form of *An. gambiae,* but was not found in the M form or *An. arabiensis* [41]. The frequent occurrence and accumulation of the *kdr* alleles in *An. sinensis* in central China may be explained by their large population size and wide distribution range. The mutant alleles may have had better chances to be selected and maintained in the *An. sinensis* populations in central China.

In this study, no *kdr* alleles were detected in all 232 specimens of the other eight member species of the *An. hyrcanus* group, including 167 specimens of *An. lesteri.* This resembles a finding reported by Kang *et al.* [42], in which the *kdr* alleles were only found in *An. sinensis*, not in the other five species (*Anopheles pullus, Anopheles kleini, An. lesteri* and *An. belenrae*) in the *An. hyrcanus* group from the Republic of Korea [42]. As a primary malaria vector in China, *An. lesteri* (previously known as *An. anthropaphagus*, which was rectified as *An. lesteri* based on the rDNA ITS2 sequences [23]), has been a major target of the vector control programmes. The extensive implementation of IRS and ITNs in 1990s-2010s has led to a remarkable reduction in *An. lesteri* abundance in China, which has resulted in a significant drop in malaria morbidity [43-45]. It is intriguing that the low *kdr* occurrence in some *An. sinensis* populations and absence in *An. lesteri* (in this study) given that both mosquitoes had been exposed to strong pyrethroid pressure. Further study is needed to investigate the resistant status and underlying mechanisms in these populations.

## Conclusions

The longitudinal survey of the historical samples revealed that the *kdr* mutations in *An. sinensis* largely occurred and accumulated progressively over time in central China. The differential *kdr* mutation distribution patterns suggest that diverse resistance mechanisms occur in different populations. Further studies are required to understand how the *kdr* alleles disperse among populations.

## Additional files

Additional file 1:
**The frequency of** ***kdr*** **alleles in the** ***Anopheles sinensis*** **samples in China.**
Additional file 2:
**The distribution of** ***kdr*** **alleles in the** ***Anopheles sinensis*** **samples in China.**
